# Gender differences in psychiatric diagnoses in older people with intellectual disability: a register study

**DOI:** 10.1186/s12888-017-1353-8

**Published:** 2017-05-22

**Authors:** Anna Axmon, Magnus Sandberg, Gerd Ahlström

**Affiliations:** 1 Division of Occupational and Environmental Medicine, Lund University, SE-221 00 Lund, Sweden; 2 Department of Health Sciences, Lund University, SE-221 00 Lund, Sweden

**Keywords:** Aged, Intellectual disability, Mental disorders, Middle aged, Sex, Sweden

## Abstract

**Background:**

Gender differences regarding psychiatric ill-health are well known in the general population. However, not much research is done on people with intellectual disability, and especially not among older people with intellectual disability.

**Methods:**

People with intellectual disability aged 55+ years in 2012 in Sweden were identified through a register containing information on those receiving support and service for this type of disability. The cohort comprised 3609 women and 4327 men with mean age 65 and 64 years, respectively. Information on psychiatric diagnoses was collected from the National Patient Register for the period 2002–2012. Potential gender differences were evaluated both for diagnostic categories (e.g. affective disorders) and single diagnoses (e.g. depressive episodes).

**Results:**

The most common diagnoses among women were in the diagnostic category affective disorders, and among men in psychotic disorders. The majority of both women (72%) and men (71%) had diagnoses in only one diagnostic category. Women were more likely than men to have at least one diagnosis of dementia (odds ratio 1.40, 95% confidence interval 1.06–1.83) or affective disorders (1.33, 1.21–1.58) during the study period. They were, however, less likely to have at least one diagnosis of alcohol/substance use related disorder (0.59, 0.43–0.80). No gender differences were found for diagnoses of psychotic (1.04, 0.86–1.27) or anxiety disorders (1.15, 0.94–1.40). Regarding single diagnoses, women were more likely than men to have had at least one diagnosis of unspecified nonorganic psychosis (1.75, 1.23–2.50), depressive episode (1.47, 1.19–1.82), recurrent depressive disorder (1.53, 1.06–2.22), other anxiety disorder (1.34, 1.06–1.69), or dementia in Alzheimer disease (2.50, 1.40–4.49), but less likely to be diagnosed with psychiatric and behavioral disorders due to use of alcohol (0.41, 0.27–0.61).

**Conclusions:**

As in the general population, there seem to be gender differences with respect to several types of psychiatric diagnoses among older people with intellectual disability. More research is needed to establish if this is due to gender differences in the occurrence of disease, inclination to seek care, health care utilization patterns, or ability to correctly identify disorders.

## Background

In the general population, there are well-known gender differences with respect to psychiatric ill-health. Whereas women are at higher risk for e.g. affective disorders [1], anxiety disorders [2], and dementia [3], men are more likely to suffer from e.g. disorders due to alcohol and drug abuse and dependence [4]. Although part of the gender differences may be attributable to biological and genetical differences [5, 6], societal factors, such as gender roles and gender equality [7, 8], and work-related gender inequality [9–11] may also be of importance. As people with intellectual disability (ID) are likely to differ from the general population with respect to societal factors, the results regarding the association between gender and psychiatric ill-health may not necessarily be applicable to this population.

Over the past decades, only few studies have focused specifically on assessing gender differences in psychiatric disorders among people with ID [12–14], and a further few have included gender as one of several potentially explanatory predictors [15–20]. The results from these studies suggest that there may be gender differences with respect to overall psychiatric disorders [13, 17] as well as some specific diagnoses and diagnostic categories [12–15, 18, 20]. However, the results are far from consistent. Thus, further investigation into this topic is needed.

Recent years have seen a steady increase in the number of people with ID who reach older age [21–23]. In the general population, the gender differences for psychiatric ill-health have been found to vary with age. For example, the increased risk of depression among women is primarily found during the early teenage years and up to menopause [24, 25]. Yet, we have found only one study, the Dutch “Healthy Ageing and Intellectual Disabilities” study [15, 20], that have investigated psychiatric diagnoses other than dementia among older people with ID specifically, and their analyses were restricted to diagnoses of depression and anxiety. Thus, more knowledge is needed to be able provide gender specific support and service for ageing people with ID.

## Methods

The aim of the present study was to describe and investigate possible gender differences in psychiatric diagnoses among older people with ID in Sweden, using data from national registers.

### Setting

In Sweden, people with ID or autism spectrum disorder (ASD) may apply to their municipality to receive one or more of ten specified services provided according to the Act Concerning Support and Service for People with Certain Functional Impairments (LSS) [26]. All such support is recorded in the national LSS-register, which is maintained by the Swedish National Board of Health and Welfare. Thanks to this, cohorts of people with one or several such service measures may be easily identified. In the present study, we have used these measures as proxy for having ID.

The Swedish National Patient Register (NPR) contains information about all in- and outpatient specialist care in Sweden. For each registration, one primary and up to 21 secondary diagnoses are listed, coded according to the 10th revision of the International Classification of Disease (ICD-10). The primary diagnosis is supposed to reflect the main reason for contact with the health care provider, whereas the secondary diagnoses give information about other conditions of relevance [27]. Thus, data regarding diagnoses of psychiatric disorders can be collected for defined cohorts of people in Sweden.

### Study population

Through the LSS register, we identified all people aged at least 55 years, with at least one service in 2012, and alive at the end of that year. This resulted in a cohort which comprised 3609 women and 4327 men. The mean age on December 31, 2012, was 65 years (range 55–96) for the women and 64 years (range 55–95) for the men.

### Outcomes

Information on psychiatric diagnoses during the study period, i.e. 2002–2012, was collected from the NPR. Diagnostic categories were chosen for investigation based on their presumed clinical relevance among older people with ID. These categories were psychotic disorders (F20-F29 in ICD-10), affective disorders (F30-F39), anxiety disorders (F40-F43), personality disorders (F60-F61), alcohol/substance use related disorders (F10-F19), and dementia (F00-F03, F05.1, F10.7A, G30-G31). Each person was categorized as having none or at least one diagnosis in each respective diagnostic category during the study period. Moreover, in order to evaluate gender differences in setting specific diagnoses, we investigated diagnoses of “unspecified mental disorder” (F99).

### Statistics

To compare the fraction of men and women with at least one diagnosis in each respective psychiatric diagnostic category and for each investigated diagnosis, we estimated odds ratios (ORs) with 95% confidence intervals (CIs) using logistic regression. Analyses were only performed if both groups to be compared contained at least 5 observations. All analyses were adjusted for year of birth. Moreover, for each diagnostic category, a possible interaction between gender and year of birth was investigated by introducing the cross-product of these to variables to the model. Wherever such an interaction was present, results are presented stratified by year of birth, trichotomized at the 33rd (1946) and 67th (1952) percentile, respectively.

A two-tailed *p*-value of 0.05 was considered statistically significant. All analyses were performed in IBM SPSS Statistics 23.

## Results

During the study period, 649 (18%) women and 725 (17%) men had at least one psychiatric diagnosis (OR for women vs men 1.11, 95% CI 0.99–1.25). Among those with at least one diagnosis, 465 women (72%) and 529 men (73%) had only one diagnosis, the most common one among women being affective disorders and among men psychotic disorders (Table 1).

**Table 1:** Diagnoses in different diagnostic categories

	Women	Men
	n (%)	n (%)
**One diagnosis**	**465 (72)**	**529** (**73**)
Affective disorder	155 (24)	138 (19)
Anxiety disorder	89 (14)	105 (14)
Dementia	87 (13)	61 (8)
Personality disorder	3 (0)	5 (1)
Psychotic disorder	108 (17)	145 (20)
Alcohol/substance use related disorder	23 (4)	75 (10)
**Two diagnoses**	**136** (**21**)	**140** (**19**)
**Three diagnoses**	**34** (**5**)	**42** (**6**)
**Four diagnoses**	**12** (**2**)	**13** (**2**)
**Five diagnoses**	**2** (**0**)	**1** (**0**)

Number and type of psychiatric diagnoses in inpatient or outpatient specialist care during 2002–2012 among 3609 women and 4327 men with intellectual disability aged 44 years or more in 2002

Women were 33% more likely than men to have been diagnosed with affective disorders and 40% more likely to have been diagnosed with dementia (Table 2). However, they only had 59% of the risk displayed by men to have been diagnosed with alcohol/substance use related disorders. No statistically significant gender differences were found for psychotic, anxiety, or personality disorders. With respect to single diagnoses, women were more likely than men to have been diagnosed with unspecified nonorganic psychosis, depressive episode, recurrent depressive disorder, other anxiety disorder, or dementia in Alzheimer disease. They were less likely to have been diagnosed with psychiatric and behavioral disorders due to use of alcohol. No statistically significant gender differences were found for any of the other investigated diagnoses.

**Table 2:** Odds ratios (ORs) with 95% confidence intervals (CIs) for women vs men for different psychiatric diagnoses

	Women	Men	Women vs men
	n (%)	n (%)	OR (95% CI)
**Psychotic disorders**	**196 (5)**	**229 (5)**	**1.04 (0.86–1.27)**
Schizophrenia (F20)	83 (2.3)	127 (2.9)	0.78 (0.59–1.04)
Schizotypal disorder (F21)	3 (0.1)	2 (0.0)	NC
Persistent delusional disorders (F22)	28 (0.8)	35 (0.8)	0.99 (0.60–1.63)
Acute and transient psychotic disorders (F23)	22 (0.6)	29 (0.7)	0.94 (0.53–1.61)
Induced delusional disorder (F24)	0 (0.0)	0 (0.0)	NC
Schizoaffective disorders (F25)	27 (0.7)	19 (0.4)	1.78 (0.99–3.21)
Other nonorganic psychotic disorders (F28)	10 (0.3)	2 (0.0)	NC
*Unspecified nonorganic psychosis (F29)	75 (2.1)	53 (1.2)	1.75 (1.23–2.50)
*Affective disorders	**297 (8)**	**279 (6)**	**1.33 (1.12–1.58)**
Manic episode (F30)	18 (0.5)	14 (0.3)	1.59 (0.79–3.21)
Bipolar affective disorder (F31)	86 (2.4)	84 (1.9)	1.24 (0.91–1.68)
*Depressive episode (F32)	193 (5.3)	165 (3.8)	1.47 (1.19–1.82)
*Recurrent depressive disorder (F33)	64 (1.8)	52 (1.2)	1.53 (1.06–2.22)
Persistent mood [affective] disorders (F34)	9 (0.2)	12 (0.3)	0.94 (0.40–2.23)
Other mood [affective] disorders (F38)	0 (0.0)	3 (0.1)	NC
Unspecified mood [affective] disorder (F39)	21 (0.6)	14 (0.3)	1.83 (0.93–3.61)
**Anxiety disorders**	**203 (6)**	**222 (5)**	**1.15 (0.94–1.40)**
Phobic anxiety disorders (F40)	19 (0.5)	17 (0.4)	1.40 (0.73–2.71)
*Other anxiety disorders (F41)	149 (4.1)	140 (3.2)	1.34 (1.06–1.69)
Obsessive-compulsive disorder (F42)	25 (0.7)	49 (1.1)	0.64 (0.39–1.04)
Reaction to severe stress, and adjustment disorders (F43)	40 (1.1)	41 (0.9)	1.24 (0.80–1.93)
**Personality disorders**	**24 (1)**	**37 (1)**	**0.81 (0.48–1.35)**
Specific personality disorders (F60)	24 (0.7)	36 (0.8)	0.83 (0.49–1.40)
Mixed and other personality disorders (F61)	0 (0.0)	2 (0.0)	NC
*Alcohol/substance use related disorders	**60 (2)**	**126 (3)**	**0.59 (0.43–0.80)**
Mental and behavioral disorders due to use of…			
* …alcohol (F10)	31 (0.9)	94 (2.2)	0.41 (0.27–0.61)
…opioids (F11)	5 (0.1)	3 (0.1)	NC
…cannabinoids (F12)	2 (0.1)	4 (0.1)	NC
…sedatives or hypnotics (F13)	6 (0.2)	2 (0.0)	NC
…cocaine (F14)	1 (0.0)	2 (0.0)	NC
…other stimulants, including caffeine (F15)	0 (0.0)	3 (0.1)	NC
…hallucinogens (F16)	0 (0.0)	0 (0.0)	NC
…tobacco (F17)	16 (0.4)	27 (0.6)	0.72 (0.39–1.34)
…volatile solvents (F18)	0 (0.0)	0 (0.0)	NC
…multiple drug use and use of other psychoactive substances (F19)	8 (0.2)	11 (0.3)	0.92 (0.37–2.30)
*Dementia	**117 (3)**	**99 (2)**	**1.40 (1.06–1.83)**
*Dementia in Alzheimer disease (F00)	34 (0.9)	17 (0.4)	2.50 (1.40–4.49)
Vascular dementia (F01)	15 (0.4)	7 (0.2)	2.43 (0.99–5.97)
Dementia in other diseases classified elsewhere (F02)	3 (0.1)	5 (0.1)	NC
Unspecified dementia (F03)	69 (1.9)	64 (1.5)	1.24 (0.88–1.75)
Delirium superimposed on dementia (F05.1)	0 (0.0)	0 (0.0)	NC
Alzheimer disease (G30)	33 (0.9)	27 (0.6)	1.53 (0.92–2.54)
Other degenerative diseases of nervous system, not elsewhere classified (G31)	5 (0.1)	6 (0.1)	0.99 (0.30–3.24)
Alcoholic dementia (F10.7A)	0 (0.0)	0 (0.0)	NC
**Unspecified mental disorder (F99)**	13 (0.4)	18 (0.4)	0.86 (0.42–1.77)

*NC* not calculated due to too few observations

Number of people with at least one diagnosis of different psychiatric disorders during 2002–2012, among 3609 women and 4327 men with intellectual disability aged 44 years or more in 2002. Odds ratios (ORs) with 95% confidence intervals (CIs) are estimated using logistic regression adjusted for year of birth. Asterisk indicates diagnoses with statistically significant differences between men and women

There was a statistical significant interaction between gender and year of birth with respect to anxiety disorders (*p* < 0.001) and alcohol/substance use related disorders (*p* = 0.009), but not for any of the other diagnostic categories (data not shown). The ORs for women vs men regarding diagnoses of anxiety disorders increased with birth cohort, with a lower OR of diagnosis for women born in or before 1946 to a higher OR for women born in or after 1953 (Table 3). For diagnoses of alcohol/substance use related disorders, the increased risk found for men when analyzing all data seemed to be driven by a risk increase among those born in or before 1952.

**Table 3:** Odds ratios (ORs) with 95% confidence intervals (CIs) for women vs men stratified by year of birth

	Women		Men		Women vs men
	Total n	With diagnosis n (%)	Total n	With diagnosis n (%)	OR (95% CI)
Anxiety disorders					
*Born in or before 1946	1407	38 (2.7)	1500	61 (4.1)	0.66 (0.43–0.99)
Born 1947–1952	1098	56 (5.1)	1372	65 (4.7)	1.08 (0.75–1.56)
*Born in or after 1953	1104	109 (9.9)	1455	96 (6.6)	1.55 (1.17–2.07)
Alcohol/substance use related disorders					
*Born in or before 1946	1407	8 (0.6)	1500	27 (1.8)	0.31 (0.14–0.69)
*Born 1947–1952	1098	18 (1.6)	1372	46 (3.4)	0.48 (0.28–0.83)
Born in or after 1953	1104	34 (3.1)	1455	53 (3.6)	0.84 (0.54–1.30)

Number of people with at least one diagnosis of different psychiatric disorders during 2002–2012, among 3609 women and 4327 men with intellectual disability aged 44 years or more in 2002, stratified by year of birth. Odds ratios (ORs) with 95% confidence intervals (CIs) are estimated using logistic regression. Asterisk indicates diagnoses with statistically significant differences between men and women

## Discussion

Among older people with ID, women were more likely than men to have a diagnosis of affective disorders and dementia, whereas men were more likely to have a diagnosis of alcohol/substance use related disorders. The majority of those with psychiatric illness were diagnosed in only one diagnostic category during the 11-year study period.

Before attempting to explain the results found, some weaknesses with the present study need to be acknowledged. Firstly, we used receiving service for ID or ASD as a proxy for having ID. One drawback with this is that the ID cohort may contain people with ASD but without ID. However, older people are not as likely as younger to have been diagnosed with ASD, and a large part of people with ASD have a combination of ID and ASD diagnoses [28]. Thus, a possible dilution of the ID cohort with people with ASD without ID should be small enough not to distort the results in any major way. Another problem that may arise from using the LSS register to define the ID cohort is that people who have ID but are coping without support would not be included in the cohort. Nevertheless, older people with ID often have parents that have passed away, which make them more dependent on the support from the society for daily life. Thus, we believe that in using the LSS register to define the ID cohort, we have included the large majority of people with ID in the older age group.

A second weakness may be the use of the NPR to identify diagnoses of psychiatric disorders, as this register does not include primary care. Thus, less severe cases of psychiatric ill-health may be lacking from our data. As a consequence, the prevalence estimates in each gender group cannot be viewed as disease prevalence, but only be used for gender comparisons. However, if men and women with the same disorder are not equally likely to receive specialist or inpatient care for this disorder, failure to include primary care diagnoses would affect the estimated gender differences in the present study. In a Canadian study among people with major depression, women were found to be less likely than men to visit specialist care and general practitioner, but more likely than men to visit a general practitioner only as opposed to no service use [29]. The authors concluded that primary care physicians might have a greater tendency to treat women and to refer men to specialist services. In addition, among people with anxiety or depression in Norway, the higher use of health care in the male part of the population was mainly evident in older groups [30]. If a similar pattern is found for older people with ID in Sweden, we would most likely underestimate the risk when it is higher for women and overestimate it when it is higher for men.

To the best of our knowledge, only one study has previously investigated gender differences in psychiatric disorders other than dementia among older people with ID. Data from the Dutch “Healthy Ageing and Intellectual Disability” were used to investigate depression and anxiety in older people with ID [15, 20]. The study is based on an administrative cohort of people aged 50+ years and receiving services and support for people with ID. As screening for depression and anxiety was made using self-reports, only people with borderline, mild, or moderate ID were included. People who had at least one score above the threshold value for one of the screening instruments were further examined with a standardized psychiatric interview. The analyses of these data revealed increased, but not statistically significantly so, risks for increased anxiety symptoms and anxiety disorders for women [20]. However, no gender differences were found for increased depressive symptoms or major depressive disorder. Those with depression or anxiety according to the standardized psychiatric interview were further assessed using the PAS-ADD [15]. The analyses based on these data did not reveal any increased risk for depression or anxiety for either gender.

Although onset of dementia occur earlier among people with ID than in the general population, it is still a disorder which becomes more common with increasing age [31, 32]. Thus, studies regarding dementia have an inherent focus on older people. While the prevalence estimate of dementia varies with the age group studied, studies performed so far suggest an increased risk of dementia among women compared with men [13, 31, 32].

Other studies regarding possible gender differences in psychiatric ill-health among people with ID have not focused specifically on older people. Some published results indicate that women are more likely to have at least one psychiatric diagnosis [13, 17], whereas others found no gender effects with respect to this outcome [12, 19]. Regarding specific diagnostic groups, previous studies indicate that women are more likely to be diagnosed with depression [12, 18, 33]. There are also some suggestions that women more often are diagnosed with schizophrenia [12] and overall psychotic disorders [34], although not all studies point in this direction [12, 13]. Similarly, men have been found more likely to have diagnoses of personality disorders [13], but these results are not consistent [12]. No studies so far have found gender differences with respect to anxiety disorders [12, 13, 16], affective disorders excluding depression [12], or alcohol/substance use related disorders [12].

In the present study, there was a pattern of increased risks of diagnoses for women in the diagnostic categories affective disorders, anxiety disorders, and dementia, as well as for the diagnoses included in these categories. The opposite was found for alcohol/substance use related disorders, whereas no pattern was found for psychotic disorders. Combining these results with previous research, the results may, at a first glance, seem inconclusive. However, it is noteworthy that there is no outcome for which different studies present opposite results. Thus, overall psychiatric diagnoses are either more common among women or do not differ between the genders. The same holds true for anxiety disorders, affective disorders/depression, dementia, and schizophrenia/psychotic disorders. The opposite, i.e. an increased risk for men or equal risk for both genders, is seen for personality disorders and alcohol/substance use related disorders. As only a few studies so far have focused on older people with ID, more information is needed to determine if the gender differences are similar for older and younger groups.

The occurrence of a diagnosis in a population is affected by the occurrence of the disorder, the inclination of the individuals to seek professional help, and the ability of professionals to correctly identify the disorder. Thus, gender differences in diagnoses would occur if there were gender differences with respect to any of these factors. Each of them is in turn a result of interaction of several underlying processes. For example, in the general population, gender differences with respect to the occurrence of affective disorders – and specifically depression – have been suggested to be associated with e.g. biological processes [6], societal factors [8], family situation [35], and work culture and circumstances [9–11]. There are indications that women are less inclined to seek care than men [29, 30]. Moreover, a number of factors that are associated with gender are also related to a person’s inclination to seek health care. For instance, in most western societies, the female part of the population tend to have lower socioeconomic status than the male, and a lower socioeconomic status has been associated with lower frequency of health care, regardless of actual psychiatric health [36]. Another example is coping strategies, the use of which may cause a person to reduce his or her need for care, and for which gender differences have been found when it comes to psychiatric health [37, 38]. Finally, if the disorder is displayed differently in women and men, such as is the case for e.g. depression [39], gender differences may arise regarding the correct identification of the disorder. As discussed above, in the present study, gender differences may also be present due to different health care utilization patterns among men and women regarding primary care vs specialist care.

## Conclusions

The present study, taken together with previous research, indicates that there may be gender differences in several types of psychiatric diagnoses. If this is due to differences in occurrence of disorder, inclination to seek care, differences in health care utilization patterns, or the ability to correctly identify disorders is yet unknown. This is important to consider when trying to achieve gender equality in health care for people in general as well as for people with ID.

## Abbreviations

ASD: Autism spectrum disorder
CI: Confidence interval
ICD-10: International classification of disease, 10th revision
ID: Intellectual disability
LSS: Act concerning support and service for people with certain functional impairments
NPR: National patient register
OR: Odds ratio
